# Bud-Derivatives, a Novel Source of Polyphenols and How Different Extraction Processes Affect Their Composition

**DOI:** 10.3390/foods9101343

**Published:** 2020-09-23

**Authors:** Federica Turrini, Dario Donno, Gabriele Loris Beccaro, Anna Pittaluga, Massimo Grilli, Paola Zunin, Raffaella Boggia

**Affiliations:** 1 Department of Pharmacy, University of Genoa, Viale Cembrano 4, 16148 Genoa, Italy; pittalug@difar.unige.it (A.P.); grilli@difar.unige.it (M.G.); zunin@difar.unige.it (P.Z.); boggia@difar.unige.it (R.B.); 2 Department of Agriculture, Forestry and Food Science, University of Torino, Largo Braccini 2, 10095 Grugliasco (TO), Italy; dario.donno@unito.it (D.D.); gabriele.beccaro@unito.it (G.L.B.)

**Keywords:** bud-derivatives, *botanicals*, polyphenols, UV-Visible spectroscopic fingerprint, chemometrics, targeted chromatographic fingerprint

## Abstract

The use of herbal food supplements, as a concentrate form of vegetable extracts, increased so much over the past years to count them among the relevant sources of dietetic polyphenols. Bud-derivatives are a category of botanicals perceived as a “new entry” in this sector since they are still poorly studied. Due to the lack of a manufacturing process specification, very different products can be found on the market in terms of their polyphenolic profile depending on the experimental conditions of manufacturing. In this research two different manufacturing processes, using two different protocols, and eight species (*Carpinus betulus* L., *Cornus mas* L., *Ficus carica* L., *Fraxinus excelsior* L., *Larix decidua* Mill., *Pinus montana* Mill., *Quercus petraea* (Matt.) Liebl., *Tilia tomentosa* Moench), commonly used to produce bud-derivatives, have been considered as a case study. An untargeted spectroscopic fingerprint of the extracts, coupled to chemometrics, provide to be a useful tool to identify these botanicals. The targeted phytochemical fingerprint by HPLC provided a screening of the main bud-derivatives polyphenolic classes highlighting a high variability depending on both method and protocol used. Nevertheless, ultrasonic extraction proved to be less sensitive to the different extraction protocols than conventional maceration regarding the extract polyphenolic profile.

## 1. Introduction

In recent decades, food supplements have an important impact on the consumers showing a significant expectation for their health and well-being [1]. They are concentrated sources of nutrients or bioactive compounds endowed with nutritional or physiological effects and, due to their presumed health benefits, they can supplement the common diet [2,3].

In particular, the interest in herbal food supplements (botanicals) is exponentially grown and consequently the relative market has increased in all the world [4]. Botanicals are become among the most popular into the food supplements category, due to the general belief which “natural” is better, healthier and safer than synthetic drugs, although this is not always true [4]. In Italy, more than 20% of the Italian population is considered “regular” consumer of these herbal products, as highlighted from the recent European PlantLibra (Plant Food Supplements: Levels of Intake, Benefit and Risk Assessment) consumer survey [5]. The wide range of herbal food supplements on the market and the non-attendance of effective legislation to guarantee the safety and quality aspects make these products vulnerable for fraud, falsification and adulteration [6,7].

Bud-derivatives (BDs) are a relatively new category of herbal food supplements and they represent one of the supply chains investigated in the FINNOVER project (Innovative strategies for the development of cross-border green supply chains), an European Interreg Alcotra Italy/France project (2017–2020) whose aim is the green innovation of several agro-industrial chains [8]. BDs are conventionally produced, according to the European Pharmacopoeia VIII edition [9], by cold maceration of the fresh meristematic tissues of trees and herbaceous plants (i.e., buds and young sprouts) using as extraction solvent mixtures of water, ethanol and glycerol [10,11]. These natural products are already marketed, and a long history of use as dietary supplements for human well-being and health is reported in traditional medicine. No health claims are yet approved by the European Food Safety Authority (EFSA) and just for some of these botanicals pharmacognostic findings supported their use as adjuvants in several diseases. In fact, some in-vitro/in-vivo biological studies for human and veterinary use have been already reported in the literature. For examples, Allio et al. (2015) investigated whether *Tilia tomentosa* bud extracts affect hippocampal Gamma-aminobutyric acid (GABA) ergic synapses [12]. In other studies, bud extracts from *Salix caprea* L. have been demonstrated to inhibit voltage gated calcium channels and catecholamines secretion in mouse chromaffin cells [13]. Moreover, different patents have also been registered on the veterinary use of bud-extracts (e.g., Composition of *Salix caprea* bud-extract and its use in the treatment of animal endometritis, patent n. TO2015A000193) [14] and several studies are carried out on several bud-derivative biological effects [15,16,17]. 

Although gemmotherapy has been used since ancient times because of the peculiar content of buds in bio-active compounds, especially polyphenols, nowadays BDs are still a little studied “niche” production [18,19]. The lack of detailed scientific information and a clear and unique regulation, as well as for the category of herbal food supplements in overall [6,7,20], it makes these products high risk and there is an increase request for efficient quality control to ensure the proper identification of the botanical source and their content [21]. 

With regards to BDs, a first problem it is accidentally confusing the raw material: fresh buds must be collected, generally from spontaneous grown, in a very limited period in the late winter and/or in the early spring, corresponding to the annual germination of the plant [18]. During this period, plants may not show their distinctive characteristics and sometimes the attribution of the botanical species may be difficult for the collector. A second problem concerns the manufacturing process and the extraction protocols whose parameters are not strictly defined, and production rules are often loose and deficient [22,23,24].

Polyphenols play key roles in plant development processes and their synthesis increases when plants are under conditions of abiotic stress, thus helping the plant to cope with environmental constraints [25]. They form an integral part of the human diet and they are very abundant in plant-based foods, such as fruits and vegetables, tea, wine, and coffee [26]. Their chemical structure is based on at least one aromatic ring with one or more hydroxyl groups, which explains their known antioxidant and anti-inflammatory properties [27]. 

In recent years, many health benefits of dietetic polyphenol supplementation have been described in humans i.e., against aging and cardiovascular disease [28,29], to prevent obesity and diabetes [30,31], to modulate human gut microbiota [32] and to improve the brain cognition skills [33,34]. This knowledge guides the choice of consumers not only towards plant foods but also towards herbal food supplements, whose polyphenol content is often even more concentrated and responsible for their bioactivity [12,13]. Nevertheless, polyphenols content is strongly influenced by the manufacturing methods whose parameters are often not strictly defined (e.g., solvent ratios in the extraction mixtures, raw material/extraction mixture ratios, extraction time) and thus they could affect the final compositions [35].

In previous articles, the polyphenolic pattern of some BDs prepared starting from different botanical species have been studied [10,11,21,36]. 

In this research, eight species spontaneously grown and commonly used to produce BDs, i.e., *Carpinus betulus* L., *Cornus mas* L., *Ficus carica* L., *Fraxinus excelsior* L., *Larix decidua* Mill., *Pinus montana* Mill., *Quercus petraea* (Matt.) Liebl., *Tilia tomentosa* Moench, have been taken into account as case study. Two different manufacturing methods, one conventional (maceration) and one innovative (direct sonication), as well as two different extraction protocols have been taken into account and the corresponding polyphenolic extracts’ profiles have been investigated.

Pulsed Ultrasound-Assisted Extraction (PUAE), according to the six principles of the green extraction [37] and the twelve principles of green chemistry [38], has been employed as an innovative technique to quickly produce BDs comparing to the long conventional maceration [39]. Even if, the positive impacts of the ultrasound-assisted extraction, i.e., reduction of the extraction time, diminution of solvent and energy used, improvement in yield and selectivity, high reproducibility, intensification of diffusion and eliminating wastes, are known in the scientific literature [40,41,42], this technique is still underused in this sector.

Moreover, two different BDs manufacturing protocols, which used different extraction mixture of solvents and different solid/liquid ratio, have also been studied to evaluate both the proper identification of the botanical species and the traceability of these vegetal products regardless of extraction method and experimental conditions.

A strategy based on the untargeted UV-Visible fingerprinting coupled to chemometrics (Principal Component Analysis—PCA) has been proposed for the screening of the polyphenolic BDs profile in order to obtain a rapid control tool [43]. Finally, HPLC methods were used to obtain a targeted chromatographic profile [7,44] of the main polyphenol classes (i.e., flavonols, benzoic acids, catechins, cinnamic acids). Polyphenols are correlated with their potential health-promoting activity [45], even if they are strongly influenced both by the methods and protocols used [35].

## 2. Materials and Methods 

### 2.1. Raw Samples 

Buds, belonging to eight different vegetable species (*Carpinus betulus* L., *Cornus mas* L., *Ficus carica* L., *Fraxinus excelsior* L., *Larix decidua* Mill., *Pinus montana* Mill., *Quercus petraea* (Matt.) Liebl., *Tilia tomentosa* Moench) were collected from plants spontaneously grown in the Turin Province (Italy) and were immediately authenticated by an agronomist. Sampling has been performed in two years (2018–2019), from February to April, during the bud break (“balsamic period”). 

Table 1 reports the geo-localization coordinates of the different collection sites and the scientific naturalistic illustrations (specifically achieved during the Finnover project) of all the eight vegetable species investigated.

### 2.2. Chemicals

MilliQ ultrapure water, obtained by means of a Millipore equipment (Bedford, MA, USA) was used throughout. All chemicals employed for the extract preparations and for the subsequent analysis were HPLC-grade. They were supplied by VWR International S.r.l (Milan, Italy) and Sigma-Aldrich (St. Louis, MO, USA). Purity of all the used standards for HPLC analysis of BDs has been reported in the Supplementary Materials (Table S1).

### 2.3. Bud-Derivatives Manufacturing Applying Two Different Methods

Fresh buds, after their collection, were immediately processed to prepare the corresponding BDs in order to minimize any degradation preserving the peculiar phytocomplex as much undamaged as possible. The manufacturing was performed both in an Italian company (Geal Pharma Turin, Italy) and by the Authors in the analytical laboratory of the University of Genoa (Department of Pharmacy). 

**Figure 1:**
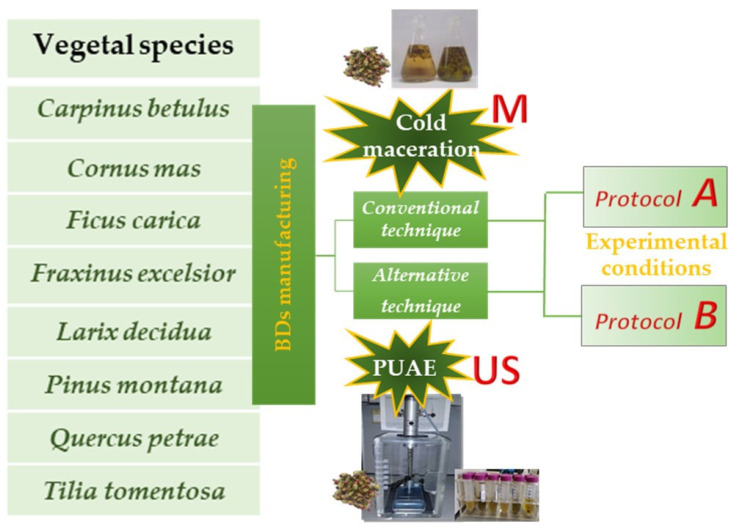
The global scheme of the experimental manufacturing of BDs: two methods (cold maceration, namely M, and ultrasounds, namely US) have been used. Each method has been applied following two different protocols (A and B).

The following two different preparation methods of BDs were investigated: the conventional cold Maceration (M) [9], and a more rapid and innovative procedure by Ultrasounds (US) recently described by the Authors [11]. Moreover, for both preparation methods two different extraction solvents and different sample/solvent ratios were investigated too (“Protocol A” and “Protocol B”, see Figure 1), in order to evaluate both the proper identification of the botanical species and the traceability of the BDs independently from their manufacturing process (Table 2). Each extraction was performed in duplicate. 

#### 2.3.1. Conventional Cold Maceration (M) as Traditional Method

BDs were prepared using a cold maceration by an Italian Company of botanicals (Geal Pharma, Bricherasio, Turin) following two different experimental manufacturing protocols, reported in Table 2:

(A) A 21 days maceration of buds in glycerol/ethanol 96% (1/1 *w*/*w*) with a 1:20 bud/solvent ratio (considering the dry weight) has been performed, according to the official method of glyceric macerates reported in the European Pharmacopoeia VIII edition [9] (“M_A”).
(B) A 3 months maceration of buds in a mixture of water/glycerol/ethanol 96% (50/20/30 *w*/*w*/*w*) as extraction solvent with a bud/solvent ratio variable (considering the fresh weight) depending on the botanical species (see Table 2) has been used, according to the method optimized and used by the Company to produce glyceric macerates (“M_B”).

In both methods, after the maceration step, the extracts, namely BDs, have been obtained by a preliminary filtration, a manual pressing and a second filtration after two days of decanting. The so obtained BDs were stored at 4 °C in the dark until their further analysis. 

#### 2.3.2. Green Extraction: Pulsed Ultrasound-Assisted Extraction (US) as Alternative Method

Fresh buds were finely ground by a Grindomix 200 M (Retsch, Haan, Germany) for 20 s at 5000 rpm, and then sieved by a 150 µm sieve, in order to improve the efficiency of the following extraction step [46]. PUAE was performed directly by an Hielscher UP200St sonicator (Teltow, Germany) equipped with an ultrasonic titanium sonotrode (7 mm of diameter), at a constant frequency of 26 kHz. The pulsed mode, referring to an alternation of “on” time and “off” time of the sonicator, guarantees a lowering increase in temperature, which better preserve the phytocomplex, and greater energy savings compared to continuous treatments [47]. The experimental sonication conditions (amplitude 30%, duty cycle 65%, extraction time 20 min) were previously optimized by the Authors on the same raw materials [11]. 

The same two experimental extraction conditions described in the paragraph 2.3.1 (“Protocol A” and “Protocol B”, see Figure 1) were employed (“US_A” and “US_B”, see Table 2). The extracts obtained were filtered for Buchner (Whatman n. 1 paper), centrifuged at 3000 rpm for 10 min and then stored at 4 °C in the dark until analysis.

### 2.4. Spectroscopic Analysis: UV-Visible Fingerprint

UV–Visible absorption spectra (200 nm–900 nm) were recorded by a spectrophotometer Agilent Cary 100 (Varian Co., Santa Clara, CO, USA) with 0.5 nm resolution, using rectangular quartz cuvettes with 1 cm path length. BDs, before the spectroscopic analysis, were suitably diluted in the corresponding extraction solvent (glycerol/ethanol 1/1 *w*/*w* or water/glycerol/ethanol 50/20/30 *w*/*w*/*w*) depending on the followed experimental protocol (“Protocol A” and “Protocol B”, respectively). Dilution was necessary to avoid signal saturation but was subsequently considered in order to make a comparison between the different spectra achieved. BDs spectra were acquired in duplicate and then averaged. The collection was performed at room temperature (25 ± 1 °C), against a blank solution represented by the corresponding extraction solvent. 

### 2.5. HPLC Analysis

In this study, effective HPLC–DAD methods were used for fingerprint analysis and phytochemical identification of samples. Four polyphenolic classes were considered: benzoic acids (ellagic and gallic acids), catechins ((+)catechin and (-)epicatechin), cinnamic acids (caffeic, chlorogenic, coumaric, and ferulic acids), and flavonols (hyperoside, isoquercitrin, quercetin, quercitrin, and rutin). Total bioactive compound content (TBCC) was determined as the sum of the most important bioactive compounds with positive effects on human organism (“multimarker approach”) [48]. 

The external standard method was used for quantitative determination of bioactive compounds. Stock solutions of cinnamic acids and flavonols with a concentration of 1.0 mg·mL^−1^ were prepared in methanol: five calibration standards were prepared by dilution with methanol; stock solutions of benzoic acids and catechins with a concentration of 1.0 mg·mL^−1^ were prepared in 95% methanol and 5% water. In this case, five calibration standards were prepared by dilution with 50% methanol–water.

An Agilent 1200 High-Performance Liquid Chromatograph coupled to an Agilent UV-Vis diode array detector (Agilent Technologies, Santa Clara, CA, USA) was used for the chromatographic analysis. Four chromatographic methods were used to separate the bioactive molecules on a Kinetex C18 column (4.6 × 150 mm, 5 μm, Phenomenex, Torrance, CA, USA). Several mobile phases were used for bioactive compound identification and UV spectra were recorded at different wavelengths, based on HPLC methods, previously tested and validated [10,40], with some modifications: (i) a solution of 10 mM KH2PO4/H3PO4 (A) and acetonitrile (B) with a flow rate of 1.5 mL·min^−1^ (method A—analysis of cinnamic acids and flavonols, gradient analysis: 5% B to 21% B in 17 min + 21% B in 3 min + 2 min of conditioning time); (ii) a solution (A) of methanol/water/formic acid (5:95:0.1 *v*/*v*/*v*) and a mix (B) of methanol/formic acid (100:0.1 *v*/*v*) with a flow rate of 0.6 mL·min^−1^ (method B—analysis of benzoic acids and catechins, gradient analysis: 3% B to 85% B in 22 min + 85% B in 1 min + 2 min of conditioning time). UV spectra were recorded at 330 nm (A); 280 nm (B).

Biomarkers were selected for their demonstrated positive healthy properties and antioxidant capacity by literature in relation to the use of this plant-derived products. All single compounds were identified in samples by comparison and combination of their retention times and UV spectra with those of authentic standards in the same chromatographic conditions. Each sample was analyzed in triplicate and results were reported as mean value ± standard deviation to assess the repeatability of the employed methods.

### 2.6. Data Analysis

#### 2.6.1. Chemometric Analysis

Multivariate data analysis has been performed by CAT (*Chemometric Agile Tool*) software, one advanced chemometric multivariate analysis tool based on R, developed by the Chemistry Group of the Italian Chemical Society [49]. 

PCA was applied as common multivariate statistical method of unsupervised pattern recognition. Its aim is extracting important information from the data and decreasing the high-dimensional dataset volume by maintaining the important information [50,51]. 

#### 2.6.2. Data Matrices Organization

A data matrix A_32,601_ consisting of 32 rows (corresponding to the BDs analyzed, 4 samples for each of the eight botanical species investigated) and 601 columns (the absorbance values in the range of 200–500 nm of the UV-Visible spectra, with 0.5 nm of resolution) was prepared and further analyzed by PCA. Standard normal variate (SNV) transform and column autoscaling were previously performed on the spectral data to remove multiplicative effects of scattering and to scale the data, respectively [52]. 

Available sample were divided in two different subsets: a calibration (or training) set and a test (or evaluation) set in order to build and validate the statistical model, respectively [53]. For a reliable validation strategy, it is important that data used as test set were not used to build the model in order to avoid the overestimations of the prediction ability [53]. 32 samples, previously reported in Table 2, were selected for the construction and identification of the model (Calibration set). The representative calibration data set consisted of 4 extracts (M_A, M_B, US_A, US_B) for each botanical species investigated (*Carpinus betulus* L., *Cornus mas* L., *Ficus carica* L., *Fraxinus excelsior* L., *Larix decidua* Mill., *Pinus montana* Mill., *Quercus petraea* (Matt.) Liebl., *Tilia tomentosa* Moench). Furthermore 16 BDs, obtained both by conventional maceration and ultrasound extraction respectively from the same eight vegetal species, were randomly selected and used as an independent set to test the model and assess its validity (Test set, Table 3). 

**Figure 2:**
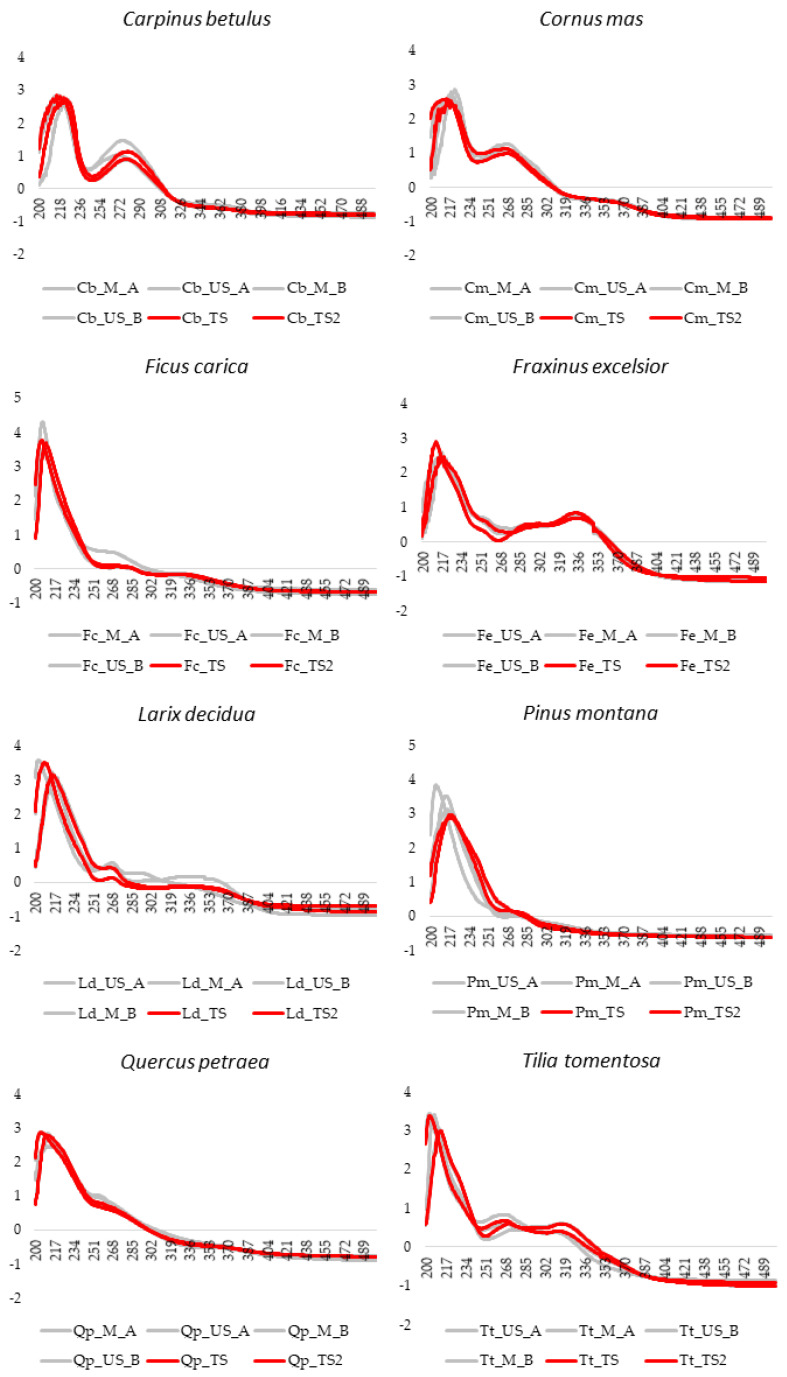
Averaged UV-Visible spectra of the 8 botanical species after SNV pre-treatment of data. For each species, the four averaged spectral profiles of the Calibration set (Table 2) are highlighted in grey while in red are reported the External Test set samples (Table 3).

All the pre-treated UV-Visible absorption spectra, in the range 200–500 nm, are reported in Figure 2. For each species, the four averaged spectral profiles corresponding to the Calibration set (Table 2) are highlighted in grey while in red have been reported the Test set samples (TS/TS2) belonging to the same class.

Then, a data matrix B_32,620_ consisting of 32 rows and 620 columns was prepared and analogously analyzed by PCA. B_32,620_ rows correspond to the 32 BDs analyzed (Calibration set), and columns are the absorbance values of the UV-Visible spectra after SNV in the range 200–500 nm coupled to the chromatographic quantifications by HPLC (4 polyphenolic classes and 13 bioactive compounds). The data set was previously scaled by using a block scaling procedure [54], with the aim to give to the spectroscopic and chromatographic variables a comparable influence in the data analysis. In fact, this pretreatment allows to divide variables in different blocks whose values will be scaled to attain the same block-variance after pretreatment. Moreover, the variables belonging to the same block are equally weighted.

## 3. Results and Discussion

The quality control of vegetal material is critical both if the botanical product is to be used as a drug or as an herbal food supplement. For consumer safety and the protection of who operate in this industrial field, quality control should be applied throughout the different processing steps, from the raw material to the final product. Scientific-naturalistic illustrations of the most common buds used in BDs production (Table 1) have been realized within the Finnover project by an expert botanical graphic designer, in order to provide a useful first tool for the operators in the BDs manufacturing. In fact, this peculiar raw material is generally spontaneously collected and mistakes in the attribution of some botanical species may be possible. For this, bud illustrations could represent a preliminary control of these vegetable materials after their collection in the point of view of a controlled manufacturing chain of BDs.

Moreover, a strategy based on the untargeted UV-Visible fingerprinting coupled to chemometrics allows rapid screening of the polyphenolic BDs profile to obtain a preliminary control tool to identify the botanical species. 

### 3.1. Bud-Derivatives Identification: UV-Visible Fingerprint

Figure 2 show the UV–Visible spectral profiles, after SNV pretreatment of the data, recorded for the eight vegetable species investigated: *Carpinus betulus* L., *Cornus mas* L., *Ficus carica* L., *Fraxinus excelsior* L., *Larix decidua* Mill., *Pinus montana* Mill., *Quercus petraea* (Matt.) Liebl., *Tilia tomentosa* Moench. The extracts were obtained by the conventional maceration and the innovative green extraction (M or US) respectively, using the two experimental protocols (A or B) as described in detail in Table 2. Ultrasounds represent one of the innovative processing techniques of officinal plants [39]. In fact, several companies already exploit innovative applications of ultrasound to obtain liquid foods, beverages, and alcoholic drinks [55,56]. Previously, the Authors described PUAE as an alternative time-saving method to the conventional maceration for the extraction of the polyphenolic fraction from buds [11]. Particularly, PUAE on a lab pilot reactor demonstrated to be an excellent approach for a rapid (20 min vs. 21 days or 3 months of maceration, depending on the Protocol applied) and efficient extraction of phenolic compounds.

Looking at Figure 2, the spectra of the different vegetable species are quite different, highlighting as the pattern of absorbances in the UV–Visible region is strictly connected with the botanical origin of the plants. On the contrary, for each botanical species the spectral differences due to the extraction method (M or US) and to the extraction solvent (Protocol A or B), are minimal. The 501–900 nm interval has been preliminarily removed because there were none interesting absorptions in this spectral region at the assayed concentrations. 

**Figure 3:**
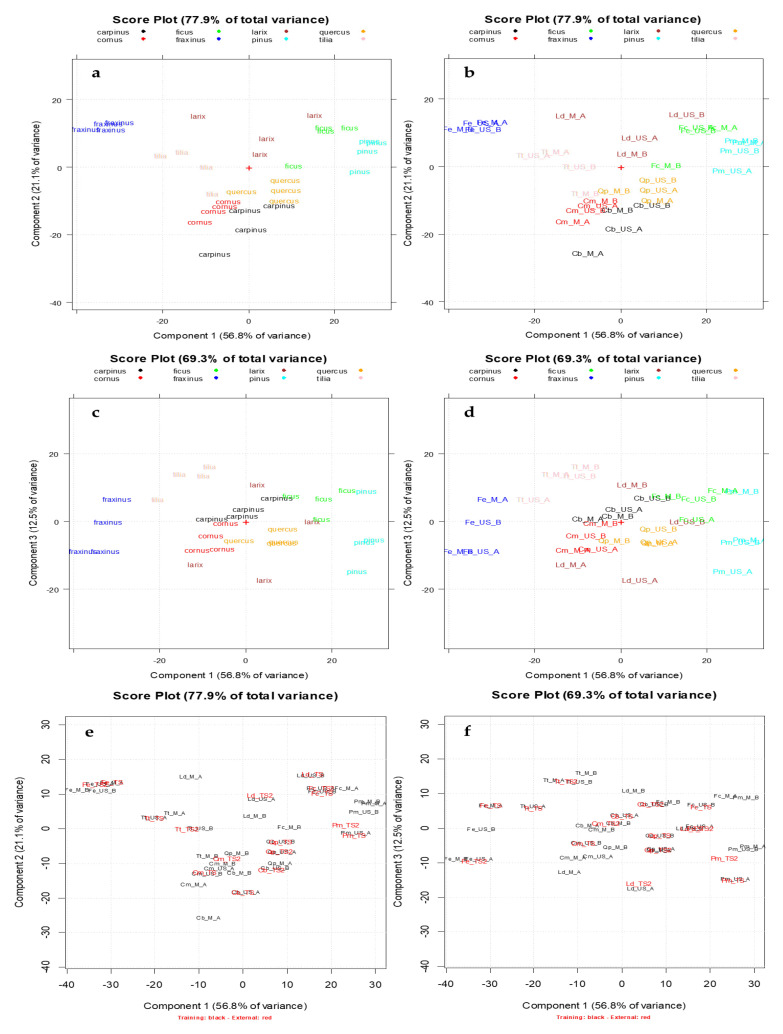
The scores plots of the UV–Visible absorbances data matrix A_32,601_. Each vegetable species is reported with a different color (*Carpinus betulus* L.: black, *Cornus mas* L.: red, *Ficus carica* L: green., *Fraxinus excelsior* L.: blue, *Larix decidua* Mill.: brown, *Pinus montana* Mill.: light blue, *Quercus petraea* (Matt.) Liebl.: orange, *Tilia tomentosa* Moench: pink). (**a**) PC1-PC2 score plot with BDs categorized according to the vegetable species; (**b**) PC1-PC2 score plot with BDs categorized according to their identification code (Table 2); (**c**) PC1-PC3 score plot with BDs categorized according to the vegetable species; (**d**) PC1-PC3 score plot with BDs categorized according to their identification code (Table 2); (**e**) PC1-PC2 score plot obtained projecting the external test set samples (highlighted in red); (**f**) PC1-PC3 score plot obtained projecting the external test set samples (highlighted in red).

PCA, an unsupervised pattern recognition technique [50,51], was applied in order to explore and to analyze the data set using a multivariate approach since the analytical information contained in each spectrum was considered as a multivariate fingerprint. Particularly, the data matrix A_32,601_, whose rows are the extracts (Calibration set) and the columns are the absorbances recorded in the spectral range 200–500 nm, was considered. PCA was performed on the pretreated and autoscaled data matrix. The first two principal components (PCs) of the data set (A_32,601_), which together explained the 77.9% of the total information of the data set since they visualize almost the 80% of the total variance, were firstly taken into account. Figure 3a,b shows the PCA score plots on the 1st–2nd principal components (PC1-PC2) obtained from the above-mentioned data matrix. In Figure 3a the extracts are categorized according to the vegetable species and each one is visualized with a different color (*Carpinus betulus* L.: black, *Cornus mas* L.: red, *Ficus carica* L: green., *Fraxinus excelsior* L.: blue, *Larix decidua* Mill.: brown, *Pinus montana* Mill.: light blue, *Quercus petraea* (Matt.) Liebl.: orange, *Tilia tomentosa* Moench: pink). In Figure 3b, for each vegetable class all the extracts belonging to the calibration set were indicated with their identification code (see Table 2). PC1, the direction of maximum variance which explains almost the 60% of the total information, allows good discrimination between the botanical class regardless of the extraction method (M or US) and the experimental preparation protocol (A or B). Particularly, the *Fraxinus* class (blue, lowest scores on PC1) separates from *Ficus* (green) and *Pinus* (light blue) which have higher scores on PC1. PC2, which explains the 21.1% of the remaining variance, allows to mainly separate *Larix* class (brown, highest scores on PC2) from *Quercus* (orange) and *Carpinus* (black, lowest scores on PC2). 

Figure 3c,d show the PCA score plots on the PC1-PC3, which explain together the 69.3% of the total variance of the data set. A good separation among the above cited botanical classes is also highlighted except for *Larix* and *Carpinus* ones. In fact, these latter separate on PC2 (Figure 3a,b) and since PCs are orthogonal, they are uncorrelated and no duplicate information are shown in their plots [50].

In Figure 3e,f, the projections of the external test set (red samples) were reported on the PC1-PC2 and PC1-PC3 score plots respectively, showing a good correspondence with the calibration set for each botanical species.

**Figure 4:**
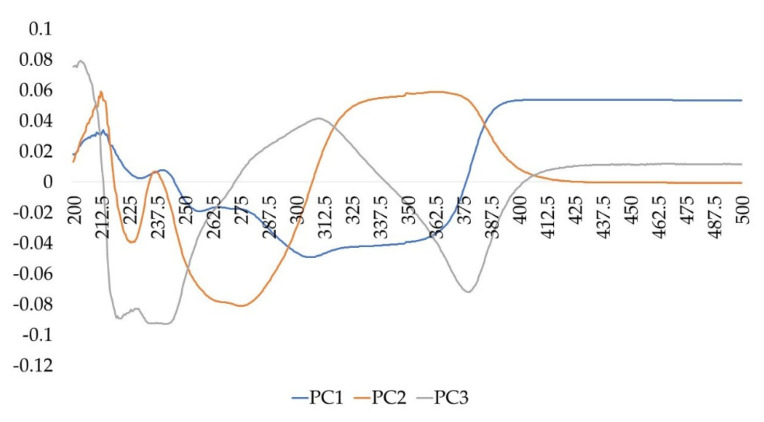
Loading plot on PC1, PC2, PC3.

The spectral variables having greater importance (loading values) on the first three PCs are represented by spectral areas near the following absorbances (in ascending order): 200 nm, 212 nm, 240 nm, 275 nm, 310 nm, 360 nm, 420 nm, as highlighted in the Loading plot on PC1-PC2-PC3 (Figure 4).

Several of them could be related to some secondary metabolites largely distributed in plant material (even in buds) such as tannins, whose structural variability depends on the vegetal species and even among organs of the same plant species [57]. The chemotaxonomic values of tannins have been recognized in the literature for several botanical species [58,59] and, the distribution of hydrolysable tannins has been used as chemotaxonomic markers by several authors [60].

It is well known that the different classes of tannins present characteristic absorption bands in the UV spectral region. Particularly as far as hydrolysable tannins are concerned, gallotannins show two characteristic absorption maximums, λ max around 212 nm and λ max around 275 nm, with an inflection point (λ min) around 242 nm; ellagitannins present strong absorption near 200 nm and a shoulder around 277 nm and another absorption near 360 nm. Instead condensed tannins (or proanthocyanidins), chemically defined as flavonoid polymers in which the phenolic hydroxyls are partially or totally esterified with gallic acid, present an absorption around 200 nm, a λ min between 258–259 nm and λ max between 279–281 nm [57]. Nevertheless, also other polyphenols, such as hydroxycinnamic acids and flavonoids, could contribute to the UV-Visible fingerprints, even if some of them are more ubiquitarians and lesser species-specific [61,62]. Furthermore, as far as flavonoids are concerned, it is important to underline that their absorptions in the Visible are almost negligible at the measured concentrations, which are instead useful to avoid saturation of the UV region.

The fingerprint UV-Visible, at least in a preliminary screening step, seems to discriminate the peculiar polyphenols composition of BDs and could be a simple and quick method to confirm the proper identification of the botanical source after the botanic check by a professional botanist. 

### 3.2. Bud-Derivatives Identification: UV-Visible and HPLC Fingerprints

**Figure 5:**
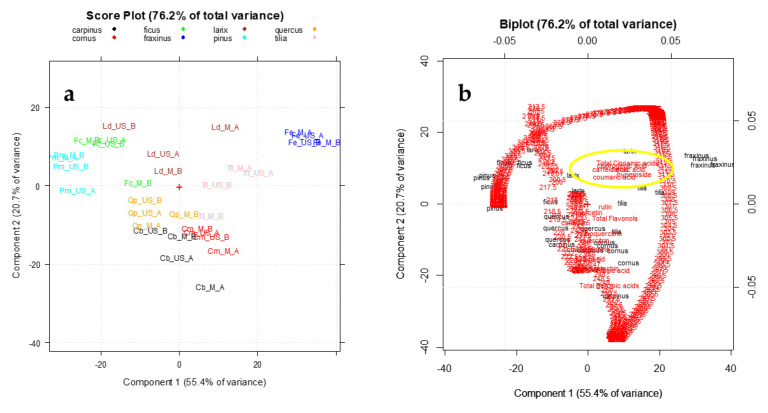
The PC1–PC2 plots of the UV–Visible absorbances coupled to the HPLC data (data matrix B_32,620_): (**a**) Score plot; (**b**) Biplot.

Figure 5 shows the PCA plots of the data matrix B_32,620_ on PC1-PC2, which together explained the 76.2% of the total variance.

PCA was performed on the pretreated and autoscaled data matrix, after the block scaling treatment in order to consider in the data analysis the same importance for the spectroscopic and chromatographic variables [63]. The PC1-PC2 score plot (Figure 5a) highlights a good separation between the vegetal species. Particularly PC1, which represents the direction of maximum variance explaining the 55.4% of the total information, allows good discrimination between *Fraxinus* class (blue, highest scores on PC1), *Ficus* (green) and *Pinus* (light blue) classes, which have lowest scores on this PC. As highlighted in the Biplot (Figure 5b) the variables having greater importance (loading value) on this separation are represented by total cinnamic acids, caffeic acid, coumaric acid and hyperoside content which are high in *Fraxinus* species and very low in *Pinus* one (as reported in Table 4). Instead PC2, which explains the 20.7% of the remaining information, allows mainly to separate *Carpinus* (black) and *Cornus* (red) classes from all the other ones. These species result particularly rich in tannins (catechins and benzoic acids).

In the Supplementary materials an example (*Larix decidua*) of chromatographic pattern was reported. As shown in Figure 5, the addition of chromatographic variables does not greatly improve the taxonomic separation previously obtained by the only UV-Visible fingerprint (Figure 3). However, these results show that the main polyphenols evaluated could be useful markers for identifying the botanical species regardless of the extraction method and the experimental preparation protocol. 

### 3.3. Phenolic Composition of BDs

**Figure 6:**
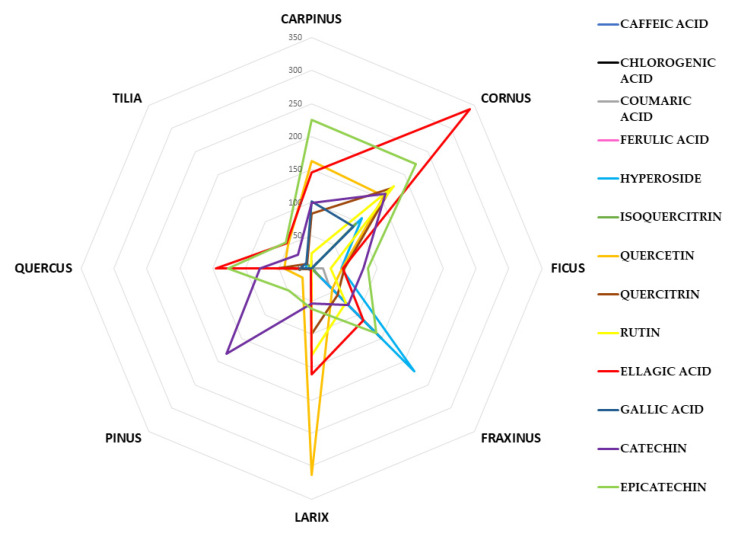
The mean content of each phenolic marker (caffeic acid, chlorogenic acid, coumaric acid, ferulic acid, hyperoside, isoquercitrin, quercetin, quercitrin and rutin, ellagic acid, gallic acid, (+)catechin and (-)epicatechin) for the eight botanical species investigated.

In this study, the health-promoting compounds were grouped into four different polyphenolic classes in order to assess the contribution of each class to the phytocomplex composition of buds belonging to the eight different species: cinnamic acids (as sum of caffeic acid, chlorogenic acid, coumaric acid, ferulic acid), flavonols (as sum of hyperoside, isoquercitrin, quercetin, quercitrin and rutin), benzoic acids (ellagic and gallic acids) and catechins ((+)catechin and (-)epicatechin). The identification and quantification of each single bioactive compound, expressed in mg/100 g _FW_, is reported in the Supplementary Materials (Table S2). For a better data visualization, Figure 6 shows the radar plot, made considering for each botanical species the mean values obtained from the 4 different extracts (M_A, M_B, US_A, US_B) for each marker compound quantified.

Several markers of cinnamic acids were considered but not detected in all the extracts. *Fraxinus excelsior* BDs showed the highest content in cinnamic acids (ranged from 113.53 ± 6.70 to 829.03 ± 2.26 mg/100 g _FW_), and as shown in Table S2, ferulic and chlorogenic acids were the most abundant. *Cornus mas* and *Tilia tomentosa* species showed very low amounts of ferulic acid (respectively 12.14 and 11.73 mg/100 g _FW_), while in the other species it was not detected. In recent years, several physiological functions of ferulic acid have been demonstrated [64]. Particularly, its free radical scavenging activity and its cholesterol-lowering activity, together with the low toxicity, suggested its chemo preventive effects on heart diseases [65]. Chlorogenic acid is also involved in beneficial effects on human health due to its anti-inflammatory, antioxidative, anti-aging and anticancer activities [66]. Chlorogenic acid was detected only in *Fraxinus excelsior* BDs (ranges from 43.88 to 489.94 mg/100 g_FW_), in all the other species it was not detectable. Li et al. 2013 reported that chlorogenic acid and flavonols may be considered the main phenolic compounds responsible for in vitro anti-cancer property (i.e., against breast, colon, liver and lung cancer) [66]. As regards the total flavonol content, it was highly variable among species. The highest content was quantified in *Cornus mas* species (mean value: 705.71 mg/100 g _FW_) while the lowest value in *Pinus montana* (mean value: 25.31 mg/100 g _FW_). As highlighted in Figure 6, quercetin represented the phenolic marker of BDs belonged to *Larix decidua* species (in orange), while hyperoside was more abundant in *Fraxinus excelsior* ones (in light blue). 

Benzoic acids are known to be very important in the human diet because of their relation to many biological and functional activities including antioxidative, anti-inflammatory, anticancer and antihepatotoxic properties [67]. Gallic acid, due to its antioxidant activity, has been shown to be effective against oxidative stress (OS), and many other properties have been reported (i.e., anti-mutagenic, anti-carcinogenic, antiviral, antibacterial, anti-inflammatory, antithrombotic and anti-atherosclerotic activities) [68]. A multi-target activity of ellagic acid, mainly ascribed to its antioxidant property and free radical trapping ability, has been reported too. In particular anti-angiogenic, anti-atherogenic, anti-carcinogenic, anti-obesity, anti-inflammatory, antioxidant, anti-thrombotic and anti-neurodegenerative properties have been demonstrated [69]. Ellagic acid was very abundant in almost all the described species (Table S2) while gallic acid was not detectable in *Ficus carica*, *Fraxinus excelsior*, *Larix decidua* and *Pinus montana* species. The highest content in ellagic acid was identified in *Cornus mas* extracts, followed by *Larix decidua* and *Quercus petraea* BDs (Figure 6, in red).

Catechins have important effects on human health thanks to its antioxidant, anti-inflammatory, antidiabetic, and antimicrobial properties [67]. The intake of foods and dietary supplements rich in catechins could have an important role in the prevention of various diseases (i.e., cardiovascular diseases), inhibition of lipid peroxidation, improvement of blood flow, elimination of several toxins and inhibition of human cancer cell line proliferation and cyclooxygenase enzymes [70]. All the vegetal species considered in this research were a good source of catechins (catechin and epicatechin) as shown in Table 3 and Table S2. Particularly, as highlighted in Figure 6, catechin represented the phenolic marker of *Pinus montana* BDs (in violet), while epicatechin was more abundant in *Carpinu betulus* and *Quercus petraea* extracts (in light green). 

**Figure 7:**
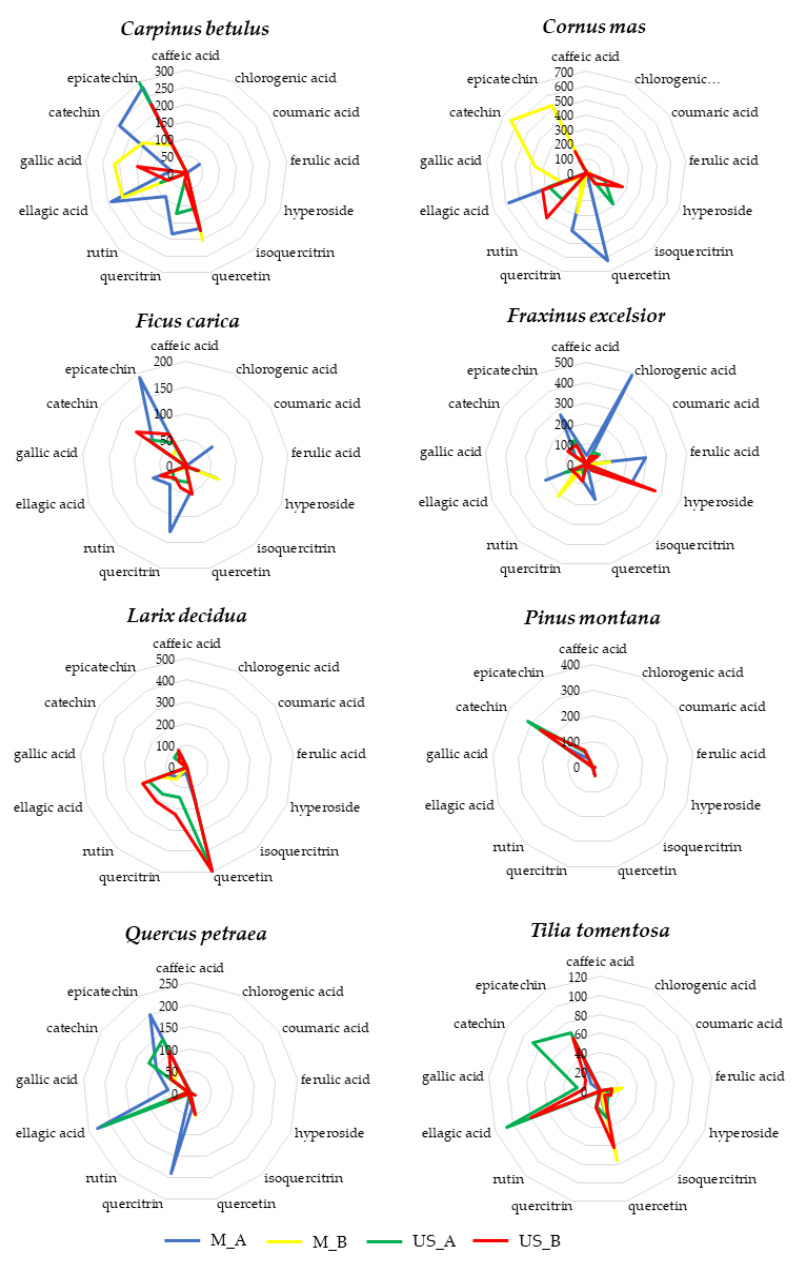
For each botanical species the phenolic composition (caffeic acid, chlorogenic acid, coumaric acid, ferulic acid, hyperoside, isoquercitrin, quercetin, quercitrin and rutin, ellagic acid, gallic acid, (+)catechin and (-)epicatechin) of the 4 different extracts (M_A: blue line, M_B: yellow line, US_A: green line, US_B: red line) was reported.

All BDs analyzed showed a good content of phenolics although there was a high variability both between the different vegetal species and between the extracts obtained by the different manufacturing method and experimental conditions starting from the same botanical species. Figure 7 showed the radar plots of each botanical species in order to better highlight the phenolic composition of the 4 different extracts (M_A, M_B, US_A, US_B).

As showed in Figure 7, the manufacturing methods (conventional maceration or sonication) and the experimental conditions used for the preparation of BDs (i.e., extraction solvent, extraction time, solid/ solvent ratio, extraction time) strongly influenced the phenolic extraction yield despite having removed the variability of the raw material (same batch of buds for each vegetal species). Generally US_A (green line) and US_B (red line) appears more similar in terms of phenolic composition respect to M extracts (M_A and M_B), except for some species, such as *Pinus montana* and *Larix decidua*, in which there is a greater homogeneity in the polyphenolic profile of the final products. In almost all species, the M_B extract (yellow line) is the most different from the others. In example, the M_B extract of *Cornus mas* was rich in catechin which was not detected in extracts obtained by different extraction conditions (M_A, US_A, US_B). Analogously, rutin represents a phenolic marker of the M_B extract of *Fraxinus excelsior*, while it was poorly detectable in the other extracts of the same species. Surely Protocol A, according to the European Pharmacopoeia, provided a higher alcoholic concentration of the extraction solvent than protocol B and it is known that a higher solvent polarity allows a higher phenolic extraction from plant materials [71]. Moreover, Protocol A used an higher solid/solvent ratio because it is evaluated on the dry weight of the raw material while following the industrial Protocol (B), the fresh weight of buds was taken into account. Regarding the effect of ultrasounds, the implosion of cavitation bubbles on the material surface results in micro-jetting which generates several effects such as surface peeling, detexturation, erosion and cell breakdown [40]. Probably, the destruction of vegetal cells allowed to increase the extraction yield making up for the lower alcohol content of protocol B. 

Due to the lack of a single regulation and an unique preparation protocol for these botanicals, very different products can be found on the market in terms of their polyphenolic fraction depending on both the raw materials (i.e., taking into account their specific agro-environmental and biological traits) and on the experimental conditions of manufacturing (method of preparation, extraction solvent, solid/solvent ratio, extraction time).

## 4. Conclusions

Although BDs have been widely used in traditional medicine because of the peculiar content of buds in phenolic compounds, nowadays they are a category of botanicals still poorly studied. The lack of detailed scientific information and a clear and unique regulation, it makes these products high risk and vulnerable for accidental mistakes in the attribution of the botanical species, but also frauds and adulterations. Moreover, the polyphenols content of BDs is strongly influenced by the manufacturing processes whose parameters are often not strictly defined (e.g., solvent ratios in the extraction mixtures, raw material/extraction mixture ratios, extraction time) and thus they affect their final compositions.

This research, within the Finnover project, aims to answer to the growing demand for efficient quality control in the BDs field to guarantee the proper attribution of the botanical source and their content. Moreover, a manufacturing process specification should be advisable to monitor the bioactive contents.

UV-Visible spectroscopy and HPLC-DAD analysis have been employed to obtain an untargeted and a targeted phytochemical fingerprint of BDs, respectively. UV-Visible coupled with an appropriate chemometric data processing is a simple, rapid and low-cost technique proved to be very useful to identify the botanical source regardless the manufacturing method and the experimental conditions used. Moreover, the targeted phytochemical fingerprint by HPLC-DAD allowed to obtain a detailed screening of the BDs polyphenolic profile which highlighted an high variability due to the different vegetal species and to the manufacturing method and protocol. The ultrasonic extraction of buds compared to conventional maceration proved less sensitive to the different extraction protocols. 

The proposed strategy offers to those operating in this industrial sector an untargeted method for the identification of the bud’s botanical species and a green extraction strategy (PUAE) which is more robust with respect to the different extractive protocols that can be used. The same approach, described for BDs, could be analogously applied to other botanical productions.

## Figures and Tables

**Table 1:** The collection sites, the corresponding geo-localization coordinates, and the scientific naturalistic illustrations of the eight different bud species.

Vegetable Species	Family (Order)	Collection Site	Geo-Localization Coordinates	Illustrations
*Carpinus betulus*	*Betulacee* *(Fagales)*	Bricherasio Prarostino San Germano Rostino	44.821, 7.285; 44.831, 7.272; 44.825, 7.275 44.913, 7.237 44.868, 7.253	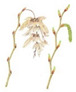
*Cornus mas*	*Cornaceae* *(Cornales)*	Bricherasio Torre Pellice Villar Pellice	44.854, 7.250; 44.855, 7.250; 44.823, 7.307 44.813, 7.181 44.804, 7.154	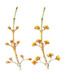
*Ficus carica*	*Moraceae* *(Rosales)*	Brondello Pagno	44.604, 7.422; 44.603, 7.419; 44.603, 7.418 44.598, 7.424; 44.597, 7.424; 44.598, 7.425	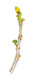
*Fraxinus excelsior*	*Oleacee* *(Lamiales)*	Angrogna Bricherasio Massello Paesana Pagno San Germano Chisone	44.869, 7.173 44.822, 7.284 44.964, 7.031 44.656, 7.261; 44.651, 7.257 44.597, 7.424; 44.598, 7.425; 44.598, 7.424 44.888, 7.261	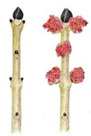
*Larix decidua*	*Pinacee* *(Pinales)*	Praly	44.902, 7.055	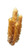
*Pinus montana*	*Pinacee* *(Pinales)*	Masello Pramollo	44.948, 7.065; 44.948, 7.068; 44.947, 7.063 44.918, 7.193	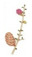
*Quercus petraea*	*Fagaceae* *(Malvales)*	Bricherasio	44.848, 7.275; 44.850, 7.274; 44.842, 7.282; 44.831, 7.270	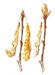
*Tilia tomentosa*	*Malvaceae* *(Malvales)*	Angrogna Bobbio Pellice Bricherasio Perrero	44.849, 7.223 44.799, 7.131 44.832, 7.265; 44.816, 7.282; 44.821, 7.273; 44.821, 7.285; 44.822, 7.283; 44.818, 7.279 44.936, 7.139	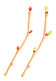

**Table 2:** BDs obtained starting from the eight vegetable species (raw materials). Two different methods (cold maceration-M and Pulsed Ultrasound-Assisted Extraction US) and two different experimental protocol (Protocol A and B) are taken into account.

	Sample Identification Code	Vegetable Species	Extraction Method	Experimental Protocol	Bud/Solvent Ratio
**1**	Cb_M_A	*Carpinus betulus*	M	Protocol A	1/20 _DW_
**2**	Cb_US_A	*Carpinus betulus*	US	Protocol A	1/20 _DW_
**3**	Cb_M_B	*Carpinus betulus*	M	Protocol B	1/15 _FW_
**4**	Cb_US_B	*Carpinus betulus*	US	Protocol B	1/15 _FW_
**5**	Cm_M_A	*Cornus mas*	M	Protocol A	1/20 _DW_
**6**	Cm_US_A	*Cornus mas*	US	Protocol A	1/20 _DW_
**7**	Cm_M_B	*Cornus mas*	M	Protocol B	1/20 _FW_
**8**	Cm_US_B	*Cornus mas*	US	Protocol B	1/20 _FW_
**9**	Fc_M_A	*Ficus carica*	M	Protocol A	1/20 _DW_
**10**	Fc _US_A	*Ficus carica*	US	Protocol A	1/20 _DW_
**11**	Fc _M_B	*Ficus carica*	M	Protocol B	1/10 _FW_
**12**	Fc_US_B	*Ficus carica*	US	Protocol B	1/10 _FW_
**13**	Fe_M_A	*Fraxinus excelsior*	M	Protocol A	1/20 _DW_
**14**	Fe_US_A	*Fraxinus excelsior*	US	Protocol A	1/20 _DW_
**15**	Fe_M_B	*Fraxinus excelsior*	M	Protocol B	1/10 _FW_
**16**	Fe_US_B	*Fraxinus excelsior*	US	Protocol B	1/10 _FW_
**17**	Ld_M_A	*Larix decidua*	M	Protocol A	1/20 _DW_
**18**	Ld_US_A	*Larix decidua*	US	Protocol A	1/20 _DW_
**19**	Ld_M_B	*Larix decidua*	M	Protocol B	1/20 _FW_
**20**	Ld_US_B	*Larix decidua*	US	Protocol B	1/20 _FW_
**21**	Pm_M_A	*Pinus montana*	M	Protocol A	1/20 _DW_
**22**	Pm_US_A	*Pinus montana*	US	Protocol A	1/20 _DW_
**23**	Pm_M_B	*Pinus montana*	M	Protocol B	1/10 _FW_
**24**	Pm_US_B	*Pinus montana*	US	Protocol B	1/10 _FW_
**25**	Qp_M_A	*Quercus petraea*	M	Protocol A	1/20 _DW_
**26**	Qp_US_B	*Quercus petraea*	US	Protocol A	1/20 _DW_
**27**	Qp_M_B	*Quercus petraea*	M	Protocol B	1/15 _FW_
**28**	Qp_US_B	*Quercus petraea*	US	Protocol B	1/15 _FW_
**29**	Tt_M_A	*Tilia tomentosa*	M	Protocol A	1/20 _DW_
**30**	Tt_US_A	*Tilia tomentosa*	US	Protocol A	1/20 _DW_
**31**	Tt_M_B	*Tilia tomentosa*	M	Protocol B	1/15 _FW_
**32**	Tt_US_B	*Tilia tomentosa*	US	Protocol B	1/15 _FW_

DW: dry weight; FW: fresh weight.

**Table 3:** External test set. 16 BDs obtained starting from the eight vegetable species using two different methods (cold maceration M and Pulsed Ultrasound-Assisted Extraction US) and two different experimental protocol (Protocol A and B) are taken into account as independent set to test the statistical model.

	Sample Identification Code	Vegetable Species	Extraction Method	Experimental Protocol
**1**	Cb_TS	*Carpinus betulus*	US	Protocol A
**2**	Cb_TS2	*Carpinus betulus*	US	Protocol B
**3**	Cm_TS	*Cornus mas*	US	Protocol A
**4**	Cm_TS2	*Cornus mas*	US	Protocol B
**5**	Fc_TS	*Ficus carica*	US	Protocol A
**6**	Fc _TS2	*Ficus carica*	US	Protocol B
**7**	Fe_TS	*Fraxinus excelsior*	M	Protocol A
**8**	Fe_TS2	*Fraxinus excelsior*	US	Protocol A
**9**	Ld_TS	*Larix decidua*	US	Protocol A
**10**	Ld_TS2	*Larix decidua*	US	Protocol B
**11**	Pm_TS	*Pinus montana*	M	Protocol A
**12**	Pm_TS2	*Pinus montana*	US	Protocol A
**13**	Qp_TS	*Quercus petraea*	M	Protocol A
**14**	Qp_TS2	*Quercus petraea*	US	Protocol A
**15**	Tt_TS	*Tilia tomentosa*	US	Protocol A
**16**	Tt_TS2	*Tilia tomentosa*	US	Protocol B

**Table 4:** Bioactive classes and total phenolics in the analyzed samples.

	Cinnamic Acids		Flavonols		Benzoic Acids		Catechins		Total Phenolics	
Sample ID	Mean Value	SD	Mean Value	SD	Mean Value	SD	Mean Value	SD	Mean Value	SD
	(mg/100 gFW **)		(mg/100 gFW **)		(mg/100 gFW **)		(mg/100 gFW **)		(mg/100 gFW **)	
Tt_M_A	5.30	0.73	51.64	2.66	22.98	0.79	52.17	1.46	132.09	5.64
Tt_M_B	23.87	1.06	90.79	5.02	6.62	1.04	50.68	1.03	171.97	8.16
Tt_US_A	5.33	1.39	71.26	5.92	132.56	1.68	156.46	1.78	365.61	10.77
Tt_US_B	12.43	5.20	100.23	14.84	96.28	8.41	81.15	10.16	290.10	38.61
Pm_M_A	n.d.	/	31.13	1.45	n.d.	/	171.38	1.65	202.51	3.10
Pm_M_B	n.d.	/	n.d.	/	n.d.	/	49.36	2.29	49.36	2.29
Pm_US_A	n.d.	/	31.36	3.86	3.67	1.56	378.90	2.54	413.93	7.96
Pm_US_B	n.d.	/	38.74	4.35	n.d.	/	325.88	4.77	364.62	9.12
Ld_M_A	n.d.	/	275.15	0.91	97.07	0.31	112.09	0.67	484.31	1.88
Ld_M_B	n.d.	/	151.57	2.23	137.23	0.88	70.90	2.62	359.70	5.72
Ld_US_A	2.40	1.02	810.86	3.32	190.25	0.95	152.12	2.12	1155.63	7.42
Ld_US_B	n.d.	/	941.62	13.22	219.28	3.66	127.08	7.33	1287.98	24.21
Fe_M_A	829.03	2.26	499.08	2.52	214.49	0.69	328.25	1.68	1870.85	7.15
Fe_M_B	119.44	0.98	223.61	3.43	40.81	1.25	98.75	2.52	482.61	8.18
Fe_US_A	151.00	2.32	378.93	4.62	115.82	0.93	225.26	2.21	871.01	10.07
Fe_US_B	113.53	6.70	551.07	10.06	77.40	2.30	215.96	5.28	957.96	24.34
Cm_M_A	23.97	0.40	1055.03	1.87	577.48	0.37	104.70	0.53	1761.19	3.18
Cm_M_B	24.59	1.55	310.99	2.06	541.34	2.35	1161.65	2.48	2038.58	8.45
Cm_US_A	14.87	1.04	672.04	3.57	276.38	1.33	98.83	1.21	1062.12	7.15
Cm_US_B	n.d.	/	784.79	12.98	329.55	2.85	167.03	4.67	1281.37	20.50
Cb_M_A	47.04	0.83	442.45	2.04	286.40	1.25	523.93	1.14	1299.83	5.26
Cb_M_B	n.d.	/	203.20	1.18	418.85	2.56	248.73	2.73	870.78	6.47
Cb_US_A	n.d.	/	230.16	2.82	80.56	1.04	297.57	1.07	608.29	4.92
Cb_US_B	n.d.	/	198.98	5.89	206.42	4.05	227.60	3.00	633.00	12.95
Fc_M_A	62.21	0.84	287.89	4.35	67.29	0.89	267.35	2.16	684.74	8.25
Fc_M_B	n.d.	/	123.28	3.65	45.86	1.08	68.42	2.11	237.57	6.83
Fc_US_A	6.49	2.62	116.68	4.31	26.33	1.18	138.27	2.64	287.77	10.76
Fc_US_B	10.77	5.54	155.02	11.39	52.18	3.49	183.91	7.34	401.88	27.76
Qp_M_A	5.08	0.65	223.63	1.97	283.59	1.28	294.75	0.85	807.06	4.75
Qp_M_B	n.d.	/	59.40	2.75	84.02	2.16	109.81	2.18	253.23	7.09
Qp_US_A	1.76	1.29	55.98	4.96	223.32	2.35	253.81	2.23	534.87	10.83
Qp_US_B	n.d.	/	72.09	8.50	58.43	5.70	161.81	4.89	292.32	19.08

SD: standard deviation; ** FW: fresh weight

## Supplementary Materials

The following are available online at https://www.mdpi.com/2304-8158/9/10/1343/s1, **Figure S1**: *Larix decidua* chromatographic pattern. Table S1: Purity of all the used standards for HPLC analysis of BDs. Table S2: Single phenolic compound fingerprint of BDs.

## References

[1] Czepielewska E., Makarewicz-Wujec M., Różewski F., Wojtasik E. (2018). Kozłowska-Wojciechowska, M. Drug adulteration of food supplements: A threat to public health in the European Union?. Regul. Toxicol. Pharmacol..

[2] Italian Ministry of Health http://www.salute.gov.it/portale/temi/p2_5.jsp?lingua=italiano&area=Alimentiparticolarieintegratori&menu=integratori.

[3] European Commission Food Supplements. https://ec.europa.eu/food/safety/labelling_nutrition/supplements_en.

[4] Colombo F., Restani P., Biella S., Di Lorenzo C. (2020). Botanicals in Functional Foods and Food Supplements: Tradition, Efficacy and Regulatory Aspects. Appl. Sci..

[5] Restani P., Di Lorenzo C., Garcia-Alvarez A., Frigerio G., Colombo F., Maggi F.M., Milà-Villarroel R., Serra-Majem L. (2018). The PlantLIBRA consumer survey: Findings on the use of plant food supplements in Italy. PLoS ONE.

[6] Deconinck E., Vanhamme M., Bothy J.L., Courselle P. (2019). A strategy based on fingerprinting and chemometrics for the detection of regulated plants in plant food supplements from the Belgian market: Two case studies. J. Pharm. Biomed. Anal..

[7] Fibigr J., Šatínský D., Solich P. (2018). Current trends in the analysis and quality control of food supplements based on plant extracts. Anal. Chim. Acta.

[8] FINNOVER Interreg Alcotra Project 2017–2020. http://www.interreg-finnover.com/.

[9] Pharmacopée Française (1965). Codex Medicamentarius Gallicus, Codex Français: Monographie, Préparations Homéopathiques.

[10] Turrini F., Donno D., Boggia R., Beccaro G.L., Zunin P., Leardi R., Pittaluga A.M. (2019). An innovative green extraction and re-use strategy to valorize food supplement by-products: Castanea sativa bud preparations as case study. Food Res. Int..

[11] Turrini F., Donno D., Beccaro G.L., Zunin P., Pittaluga A., Boggia R. (2019). Pulsed Ultrasound-Assisted Extraction as an Alternative Method to Conventional Maceration for the Extraction of the Polyphenolic Fraction of Ribes nigrum Buds: A New Category of Food Supplements Proposed by The FINNOVER Project. Foods.

[12] Allio A., Calorio C., Franchino C., Gavello D., Carbone E., Marcantoni A. (2015). Bud extracts from Tilia tomentosa Moench inhibit hippocampal neuronal firing through GABAA and benzodiazepine receptors activation. J. Ethnopharmacol..

[13] Calorio C., Donno D., Franchino C., Carabelli V., Marcantoni A. (2017). Bud extracts from Salix caprea L. inhibit voltage gated calcium channels and catecholamines secretion in mouse chromaffin cells. Phytomedicine.

[14] Nervo T., Bergamini L., Guido M., Ferraro F. (2015). Composizione e Relativo Uso Nel Trattamento Dell’endometrite Animale. U.S. Patent.

[15] Olivero G., Turrini F., Vergassola M., Boggia R., Zunin P., Donno D., Beccaro G.L., Grilli M., Pittaluga A. (2019). The 3Rs: Reduction and refinement through a multivariate statistical analysis approach in a behavioural study tounveil anxiolytic effects of natural extracts of Tilia tomentosa. Biomed. Sci. Eng..

[16] Antonaci I. (2017). Effetto Di Un Trattamento Fitoterapico Su Alcuni Parametri Ematologici Dell’asina Da Latte. Bachelor’s Thesis.

[17] Guerra C., Nury C. (2015). Utilizzo Di Una Soluzione Fitoterapica Per Un Trattamento Alternativo Dell’endometrite Equina. Bachelor’s Thesis.

[18] Donno D., Beccaro G.L., Cerutti A.K., Mellano M.G., Bounous G., Rao A.V., Rao L.G. (2015). Bud Extracts as New Phytochemical Source for Herbal Preparations-Quality Control and Standardization by Analytical Fingerprint. Phytochemicals—Isolation, Characterisation and Role in Human Health.

[19] Donno D., Mellano M.G., Cerutti A.K., Beccaro G.L. (2016). Biomolecules and Natural Medicine Preparations: Analysis of New Sources of Bioactive Compounds from *Ribes* and *Rubus* spp. Buds. Pharmaceuticals.

[20] Sanzini E., Badea M., Dos Santos A., Restani P., Sievers H. (2011). Quality control of plant food supplements. Food Funct..

[21] Donno D., Boggia R., Zunin P., Cerutti A.K., Guido M., Mellano M.G., Prgomet Z., Beccaro G.L. (2016). Phytochemical fingerprint and chemometrics for natural food preparation pattern recognition: An innovative technique in food supplement quality control. J. Food Sci. Technol..

[22] Directive 2002/46/EC of the European Parliament and of the Council of 10 June 2002 on the Approximation of the Laws of the Member States Relating to Food Supplements. https://eur-lex.europa.eu/eli/dir/2002/46/2017-07-26.

[23] Decreto Legislativo 21 Maggio 2004, n.169, Attuazione Della Direttiva 2002/46/CE Relativa Agli Integratori Alimentari. https://www.gazzettaufficiale.it/eli/id/2004/07/15/004G0201/sg.

[24] European Federation of Associations of Health Product Manufacturers (EHPM) https://www.ehpm.org/attachments/article/117/EHPM%20Quality%20Guide%20101214.pdf.

[25] Watson R.R. (2018). Polyphenols in Plants: Isolation, Purification and Extract Preparation.

[26] Ma G., Chen Y. (2020). Polyphenol supplementation benefits human health via gut microbiota: A systematic review via meta-analysis. J. Funct. Foods.

[27] Tsao R. (2010). Chemistry and biochemistry of dietary polyphenols. Nutrients.

[28] Tresserra-Rimbau A., Rimm E.B., Medina-Remón A., Martínez-González M.A., de la Torre R., Corella D., Salas-Salvadó J., Gómez-Gracia E., Lapetra J., Arós F. (2014). Inverse association between habitual polyphenol intake and incidence of cardiovascular events in the PREDIMED study. Nutr. Metab. Cardiovasc. Dis..

[29] Kwok C.S., Boekholdt S.M., Lentjes M.A.H., Loke Y.K., Luben R.N., Yeong J.K., Wareham N.J., Myint P.K., Khaw K.T. (2015). Habitual chocolate consumption and risk of cardiovascular disease among healthy men and women. Heart.

[30] Wang P.Y., Fang J.C., Gao Z.H., Zhang C., Xie S.Y. (2016). Higher intake of fruits, vegetables or their fiber reduces the risk of type 2 diabetes: A meta-analysis. J. Diabetes Investig..

[31] Wang S., Moustaid-Moussa N., Chen L., Mo H., Shastri A., Su R., Bapat P., Kwun I., Shen C.L. (2014). Novel insights of dietary polyphenols and obesity. J. Nutr. Biochem..

[32] Serra D., Almeida L.M., Dinis T.C.P. (2018). Dietary polyphenols: A novel strategy to modulate microbiota-gut-brain axis. Trends Food Sci. Technol..

[33] Vauzour D. (2012). Dietary polyphenols as modulators of brain functions: Biological actions and molecular mechanisms underpinning their beneficial effects. Oxidative Med. Cell. Longev..

[34] Liu X., Du X., Han G., Gao W. (2017). Association between tea consumption and risk of cognitive disorders: A dose-response meta-analysis of observational studies. Oncotarget.

[35] Brglez Mojzer E., Knez Hrnčič M., Škerget M., Knez Ž., Bren U. (2016). Polyphenols: Extraction Methods, Antioxidative Action, Bioavailability and Anticarcinogenic Effects. Molecules.

[36] Donno D., Beccaro G.L., Mellano M.G., Bonvegna L., Bounous G. (2014). Castanea spp. buds as a phytochemical source for herbal preparations: Botanical fingerprint for nutraceutical identification and functional food standardization. J. Sci. Food Agric..

[37] Chemat F., Vian M.A., Cravotto G. (2012). Green extraction of natural products: Concept and principles. Int. J. Mol. Sci..

[38] Green Chemistry’s 12 Principles, United States Environmental Protection Agency. https://www.epa.gov/greenchemistry/basics-green-chemistry#twelve.

[39] Ministero Delle Politiche Agricole Alimentari E Forestali Piano Di Settore Della Filiera Delle Piante Officinali 2014–16. https://www.politicheagricole.it/flex/cm/pages/ServeBLOB.php/L/IT/IDPagina/7562.

[40] Chemat F., Rombaut N., Sicaire A.G., Meullemiestre A., Fabiano-Tixier A.S., Abert-Vian M. (2017). Ultrasound assisted extraction of food and natural products. Mechanisms, techniques, combinations, protocols and applications. A review. Ultrason. Sonochem..

[41] Vinatoru M., Mason T.J., Calinescu I. (2017). Ultrasonically assisted extraction (UAE) and microwave assisted extraction (MAE) of functional compounds from plant materials. Trends Anal. Chem..

[42] Vernès L., Vian M., Chemat F., Poole C.F. (2020). Chapter 12—Ultrasound and Microwave as Green Tools for Solid-Liquid Extraction. Handbooks in Separation Science, Liquid-Phase Extraction.

[43] Boggia R., Turrini F., Anselmo M., Zunin P., Donno D., Beccaro G.L. (2017). Feasibility of UV-VIS-Fluorescence Spectroscopy combined with pattern recognition techniques to authenticate a new category of plant food supplements. J. Food Sci. Technol..

[44] Donno D., Mellano M.G., Riondato I., De Biaggi M., Andriamaniraka H., Gamba G., Beccaro G.L. (2019). Traditional and Unconventional Dried Fruit Snacks as a Source of Health-Promoting Compounds. Antioxidants.

[45] Li A.N., Li S., Zhang Y.J., Xu X.R., Chen Y.M., Li H.B. (2014). Resources and Biological Activities of Natural Polyphenol. Nutrients.

[46] Vilkhu K., Mawson R., Simons L., Bates D. (2008). Applications and opportunities for ultrasound assisted extraction in the food industry—A review. Innov. Food Sci. Emerg. Technol..

[47] Pan Z., Qu W., Ma H., Atungulu G.G., McHugh T.H. (2011). Continuous and pulsed ultrasound-assisted extractions of antioxidants from pomegranate peel. Ultrason. Sonochem..

[48] Mok D.K.W., Chau F.T. (2006). Chemical information of Chinese medicines: A challenge to chemist. Chemom. Intell. Lab. Syst..

[49] Italian Chemical Society Division of Analytical Chemistry-Group of Chemometrics. CAT Chemometric Agile Tool. http://www.gruppochemiometria.it/index.php/software.

[50] Wold S., Esbensen K., Geladi P. (1987). Principal Component Analysis. Chemom. Intell. Lab. Syst..

[51] Jolliffe I.T. (2002). Principal Component Analysis.

[52] Barnes R.J., Dhanoa M.S., Lister S.J. (1989). Standard normal variate transformation and de-trending of near-infrared diffuse reflectance spectra. Appl. Spectrosc..

[53] Oliveri P. (2017). Class-modelling in food analytical chemistry: Development, sampling, optimisation an validation issues—A tutorial. Anal. Chim. Acta.

[54] Wold S., Johansson E., Cocchi M., Hugo K. (1993). 3D QSAR in Drug Design: Theory, Methods and Applications.

[55] Chemat F., Ashokkumar M. (2017). Preface: Ultrasound in the processing of liquid foods, beverages and alcoholic drinks. Ultrason. Sonochem..

[56] Paniwnyk L. (2017). Applications of ultrasound in processing of liquid foods: A review. Ultrason. Sonochem..

[57] Falcão L., Araújo M.E.M. (2018). Vegetable Tannins Used in the Manufacture of Historic Leathers. Molecules.

[58] Okuda T., Yoshida T., Hatano T., Iwasaki M., Kubo M., Orime T., Yoshizaki M., Naruhashi N. (1992). Hydrolysable tannins as chemotaxonomic markers in the rosaceae. Phytochemistry.

[59] Yoshida T., Amakura Y., Yoshimura M. (2010). Structural features and biological properties of ellagitannins in some plant families of the order Myrtales. Int. J. Mol. Sci..

[60] Moilanen J., Koskinen P., Salminen J.P. (2015). Distribution and content of ellagitannins in Finnish plant species. Phytochemistry.

[61] Rocha L.D., Monteiro M.C., Teodoro A.J. (2012). Anticancer properties of hydroxycinnamic acids—A Review. Cancer Clin. Oncol..

[62] Zelber-Sagi S., Salomone F., Mlynarsky L. (2017). The Mediterranean dietary pattern as the diet of choice for non-alcoholic fatty liver disease: Evidence and plausible mechanisms. Liver Int..

[63] Brereton R.G. (2007). Applied Chemometrics for Scientists.

[64] De Biaggi M., Donno D., Mellano M.G., Gamba G., Riondato I., Rakotoniaina E.N., Beccaro G.L. (2020). Emerging species with nutraceutical properties: Bioactive compounds from Hovenia dulcis pseudofruits. Food Chem..

[65] Kim H.K., Jeong T.-S., Lee M.-K., Park Y.B., Choi M.-S. (2003). Lipid-lowering efficacy of hesperetin metabolites in high-cholesterol fed rats. Clin. Chim. Acta.

[66] Li F., Li S., Li H.-B., Deng G.-F., Ling W.-H., Wu S., Xu X.-R., Chen F. (2013). Antiproliferative activity of peels, pulps and seeds of 61 fruits. J. Funct. Foods.

[67] Beccaro G.L., Donno G., Lione G.G., De Biaggi M., Gamba G., Rapalino S., Riondato I., Gonthier P., Mellano M.M. (2020). Castanea spp. Agrobiodiversity Conservation: Genotype Influence on Chemical and Sensorial Traits of Cultivars Grown on the Same Clonal Rootstock. Foods.

[68] Alfei S., Signorello M.G., Schito A., Catena S., Turrini F. (2019). Reshaped as polyester-based nanoparticles, gallic acid inhibits platelet aggregation, reactive oxygen species production and multi-resistant Gram-positive bacteria with an efficiency never obtained. Nanoscale Adv..

[69] Alfei S., Turrini F., Catena S., Zunin P., Grilli M., Pittaluga A.M., Boggia R. (2019). Ellagic acid a multi-target bioactive compound for drug discovery in CNS? A narrative review. Eur. J. Med. Chem..

[70] Ananingsih V.K., Sharma A., Zhou W. (2013). Green tea catechins during food processing and storage: A review on stability and detection. Food Res. Int..

[71] Franco D., Sineiro J., Rubilar M., Sánchez M., Jerez M., Pinelo M., Costoya N., Núñez M.J. (2008). Polyphenols from plant materials: Extraction and antioxidant power. Electron. J. Environ. Agric. Food Chem..

