# Supplementary material for: Bud-Derivatives, a Novel Source of Polyphenols and How Different Extraction Processes Affect Their Composition
Source: Foods. 2020 Sep 23;9(10):1343. doi: 10.3390/foods9101343 (PMC7598208; doi:10.3390/foods9101343)
Supplement: Supplementary file 1 [file foods-09-01343-s001.zip › SUPPLEMENTARY MATERIALS/Supplementary materials Table S1.pdf]

| Chemical classes | Compound | Purity (%) |
|---|---|---|
| **Cinnamic acids** | Caffeic acid | ≥98% |
| | Chlorogenic acid | ≥95% |
| | Coumaric acid | ≥98% |
| | Ferulic acid | ≥99% |
| **Flavonols** | Hyperoside | ≥97% |
| | Isoquercitrin | ≥98% |
| | Quercetin | ≥95% |
| | Quercitrin | ≥98% |
| | Rutin | ≥95% |
| **Benzoic acids** | Ellagic acid | ≥95% |
| | Gallic acid | ≥97% |
| **Catechins** | Catechin | ≥97% |
| | Epicatechin | ≥98% |
