# Supplementary figures and images for: Bud-Derivatives, a Novel Source of Polyphenols and How Different Extraction Processes Affect Their Composition
Source: Foods. 2020 Sep 23;9(10):1343. doi: 10.3390/foods9101343 (PMC7598208; doi:10.3390/foods9101343)

**Cinnamic acids - Flavonols**

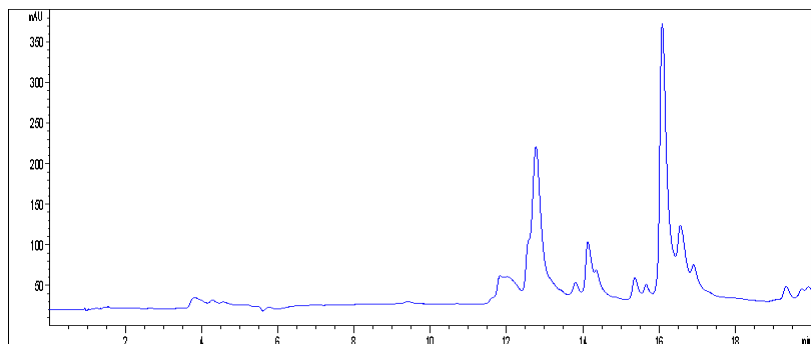

*Ld\_M\_A*

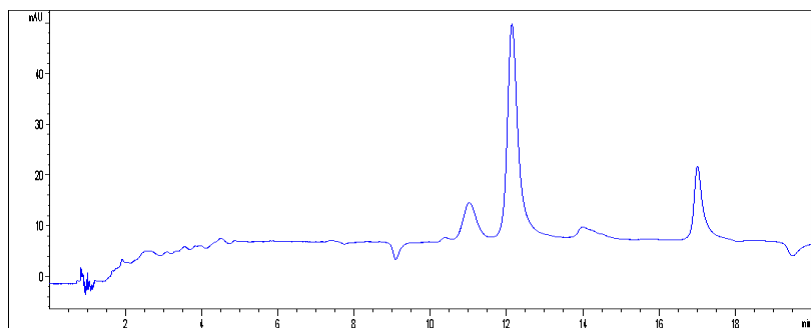

*Ld\_M\_B*

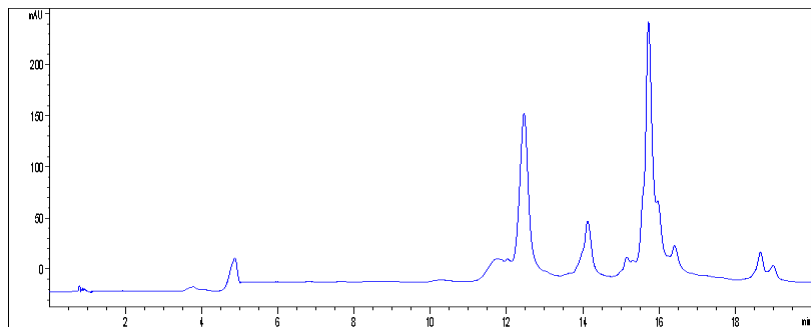

*Ld\_US\_A*

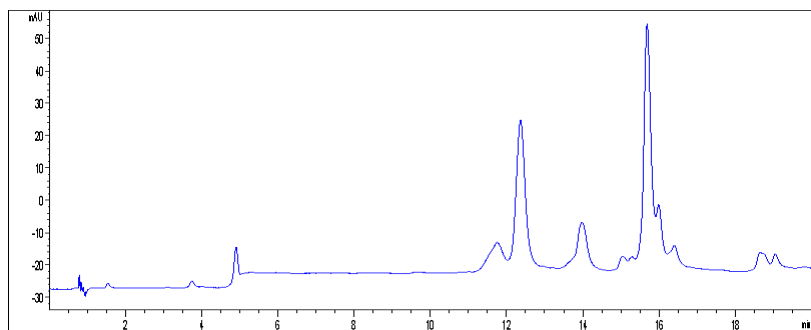

*Ld\_US\_B*

**Benzoic acids - Catechins**

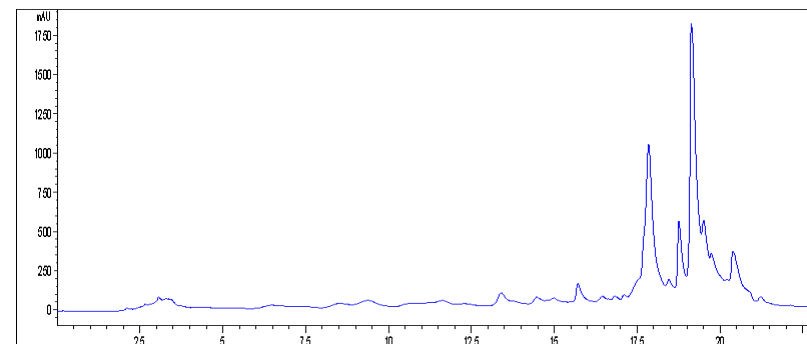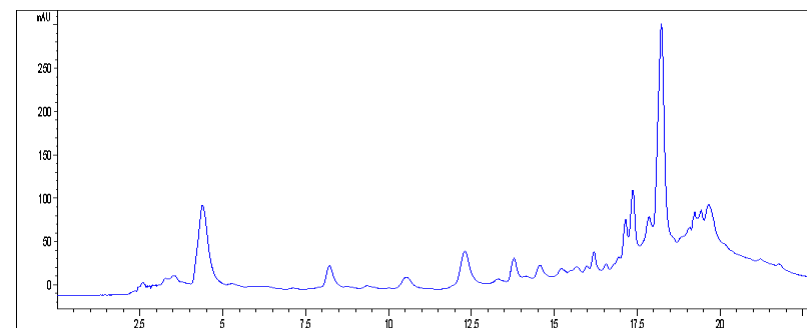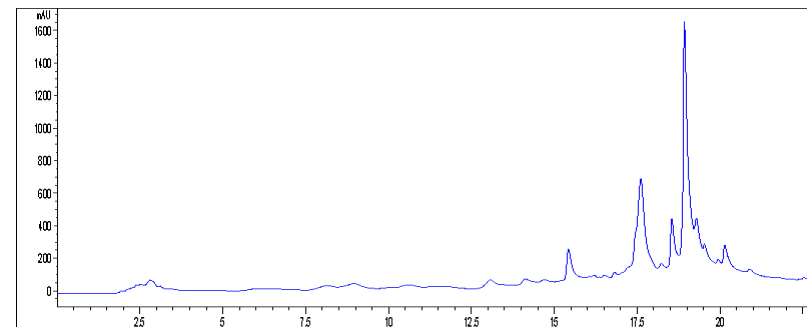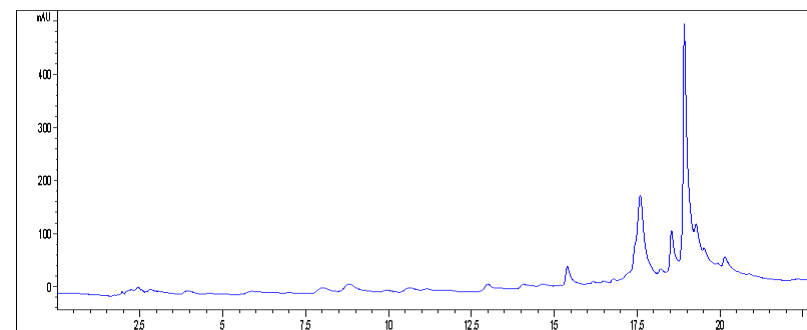

Supplement: Supplementary file 1 [file foods-09-01343-s001.zip › SUPPLEMENTARY MATERIALS/Supplementary materials Figure S1.pdf]
